# A meta-analysis comparing the effectiveness and safety of repetitive transcranial magnetic stimulation versus theta burst stimulation for treatment-resistant depression

**DOI:** 10.3389/fpsyt.2024.1504727

**Published:** 2025-02-03

**Authors:** Xiao Tao, Zheng Wen Jing, Wang Kui Yuan, Guo Hui Yun, Xie Jian Fang, Liao Ming Sheng

**Affiliations:** Department of Psychiatry, The Third People’s Hospital of Ganzhou, Ganzhou, Jiangxi, China

**Keywords:** transcranial magnetic stimulation, theta burst stimulation, treatment-resistant depression, meta-analysis, depression

## Abstract

**Objective:**

This study compares the safety and effectiveness of theta-burst stimulation (TBS) and repetitive transcranial magnetic stimulation (rTMS) for treating treatment-resistant depression (TRD).

**Methods:**

We reviewed randomized controlled trials (RCTs) that evaluated rTMS and TBS in managing TRD. Searches were conducted in PubMed, Embase, the Cochrane Library, and Web of Science for studies published up to July 31, 2024. Data from these studies were analyzed using statistical software.

**Results:**

Five RCTs involving 1,196 patients were included, with 553 receiving rTMS and 663 receiving TBS. The analysis found no significant differences between rTMS and TBS in reducing depression [SMD = -0.07, 95% CI (-0.19, 0.04)] or anxiety [SMD = -0.02, 95% CI (-0.15, 0.11)], nor in side effects like headaches [OR = 1.00, 95% CI (0.72, 1.40)], nausea [OR = 1.42, 95% CI (0.79, 2.54)], or fatigue [OR = 0.87, 95% CI (0.46, 1.64)].

**Conclusions:**

Both rTMS and TBS are similarly effective in reducing depression and anxiety symptoms, with comparable side effect profiles. However, TBS is more time-efficient, with sessions lasting only 192 seconds, making it a cost-effective option for patients. These findings support TBS as a practical treatment choice for TRD.

## 1 Introduction

Major depressive disorder (MDD) is a significant global public health concern, characterized by high morbidity, a high incidence of suicide, and a high recurrence rate (1, 2). Selective serotonin reuptake inhibitors (SSRIs) are the cornerstone of current MDD therapy. However, studies show that approximately 44% of patients who complete a full course of antidepressant treatment fail to achieve remission, leading to a prolonged depressive state (3) and ultimately resulting in treatment-resistant depression (TRD). Research indicates that about one-third of patients with TRD attempt suicide at least once in their lifetime (4, 5), severely impairing social functioning, increasing societal burdens, and posing a significant challenge in clinical practice (6–8).

TRD is typically defined as depression that does not respond to a full course of treatment with two or more antidepressants (9). Conventional pharmacological treatments often show limited efficacy in TRD, with delayed onset of therapeutic effects, significant cognitive side effects, and low remission rates, all of which contribute to poor medication adherence (10, 11). In light of these limitations, recent research has emphasized the importance of exploring alternative and multimodal strategies to address the complexity of TRD. Approaches such as augmentation with atypical antipsychotics, mood stabilizers, and agents targeting non-monoaminergic systems have demonstrated potential benefits (12, 13). For instance, cariprazine, an atypical antipsychotic, has shown efficacy as an augmentation agent in TRD, particularly in patients who failed previous augmentation trials. Additionally, treatments like esketamine nasal spray provide rapid-acting options by targeting the glutamate pathway, further underscoring the need for innovative interventions in TRD management.One promising alternative for the treatment of TRD is rTMS (14). rTMS is a relatively new brain stimulation method that has shown potential in several studies (6, 15). Its use for the treatment of TRD has been approved by Health Canada (2002), the US Food and Drug Administration (2008), and regulatory bodies in the EU, Australia, Israel (16), and other regions.

A more recent form of rTMS is TBS, a sophisticated non-invasive neuromodulation technique with a distinct stimulation pattern. Compared to traditional rTMS, TBS offers several advantages, including lower stimulation intensity, shorter session duration, better tolerability, and a closer approximation to natural neuronal activity. TBS can induce stronger and more sustained cortical excitability, thereby reducing the overall treatment duration and producing faster antidepressant effects (17). Despite these advantages, the relative effectiveness of rTMS versus TBS in treating TRD remains a topic of ongoing debate (18). This study aims to address this issue through a meta-analysis, providing professionals with clearer recommendations and offering patients more effective treatment options.

## 2 Methods

### 2.1 Systematic review registration

This systematic review has been officially registered in the PROSPERO database, an international registry of prospective systematic reviews of health-related interventions produced by the National Institute for Health Research (19).

### 2.2 Inclusion and exclusion criteria

The study population consisted of individuals diagnosed with treatment-resistant depression (TRD). The experimental group received repetitive transcranial magnetic stimulation (rTMS), while the control group was treated with theta-burst stimulation (TBS). The primary outcomes measured were anxiety and depression levels, with adverse event rates as secondary outcomes. Only randomized controlled trials (RCTs) were included. Exclusion criteria applied to meeting abstracts, meta-analyses, systematic reviews, animal studies, studies with inaccessible full text, case reports, and research involving participants who had previously undergone other treatments.

### 2.3 Literature search

A comprehensive search was conducted across the PubMed, Embase, Cochrane Library, and Web of Science databases. Keywords such as “TBS,” “rTMS,” and “TRD” were used both as free-text terms and indexed phrases. The final search update occurred on July 31, 2024. The complete search strategy is outlined in **Supplementary Table S1** in the **Supplementary Material**.

### 2.4 Data extraction

Two authors independently screened the literature based on predefined inclusion and exclusion criteria. Any disagreements were resolved through discussion, and if necessary, a third reviewer was consulted to reach a consensus. Key information extracted from the eligible studies included study characteristics, average age, sex distribution, sample size, publication year, intervention methods, and outcomes.

### 2.5 Bias risk assessment

The bias in the included studies was assessed independently by two reviewers using the Cochrane Collaboration’s methods (20). A third reviewer was consulted to resolve any disagreements. The assessment covered seven domains: completeness of outcome data (attrition bias), allocation concealment (selection bias), blinding of participants and personnel (performance bias), blinding of outcome assessors (detection bias), selective reporting (reporting bias), and other potential sources of bias. Among these, the most common biases identified were performance bias, due to inadequate blinding of participants and personnel, and detection bias, arising from the lack of blinding of outcome assessors. These biases could potentially lead to overestimation or underestimation of treatment effects, influencing the reliability and validity of the study outcomes. In particular, performance bias may result in differences in care or treatment between groups, while detection bias can affect the accuracy of outcome measurements, leading to biased conclusions about the effectiveness of interventions.

Each study was evaluated based on these criteria. Studies that met all the requirements were classified as having “low risk of bias,” indicating high quality and minimal risk. Studies that did not meet the criteria were labeled as having “high risk,” suggesting significant bias and lower quality. Those that partially met the criteria were categorized as having “unclear risk,” indicating a moderate risk of bias.

### 2.6 Data analysis

Data were statistically analyzed using Stata 15.0 (Stata Corp., College Station, TX, USA). Heterogeneity among the included studies was assessed using the Q-statistic and the I²-statistic. I² values were interpreted as follows: 25% indicated low heterogeneity, 50% indicated moderate heterogeneity, and 75% indicated high heterogeneity. If the I² value was 50% or higher, sensitivity analysis was performed to explore potential sources of heterogeneity. For I² values below 50%, a fixed effects model (FEM) was applied. For continuous variables, SMDs and 95% CIs were calculated, while ORs and 95% CIs were used for dichotomous variables. Additionally, Egger’s test and a random-effects model (REM) were applied to assess publication bias.

## 3 Results

### 3.1 Literature search

**Figure 1:**
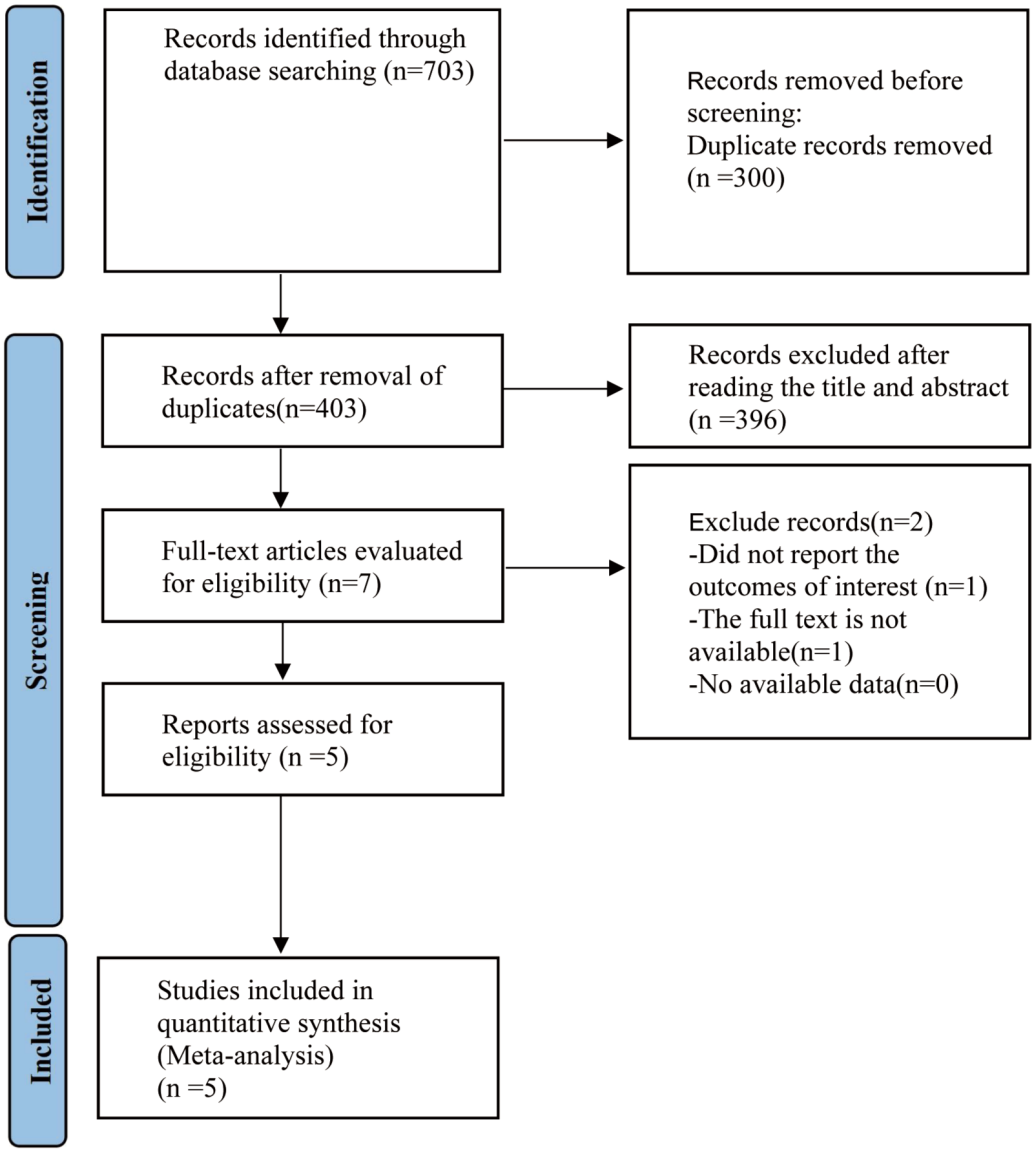
RISMA diagram of research procedure. PRISMA, preferred reporting items for systemic review and meta-analysis.

**Figure 1** illustrates the methods used for the literature search. A total of 703 articles were identified from PubMed (n = 115), Embase (n = 162), the Cochrane Library (n = 158), and Web of Science (n = 268). After removing 300 duplicates and excluding 396 articles based on titles and abstracts, two additional articles were eliminated after full-text review. Ultimately, five randomized controlled trials (RCTs) (21–25) were included in the study.

### 3.2 Baseline features and bias risk in associated research 

**Figure 2:**
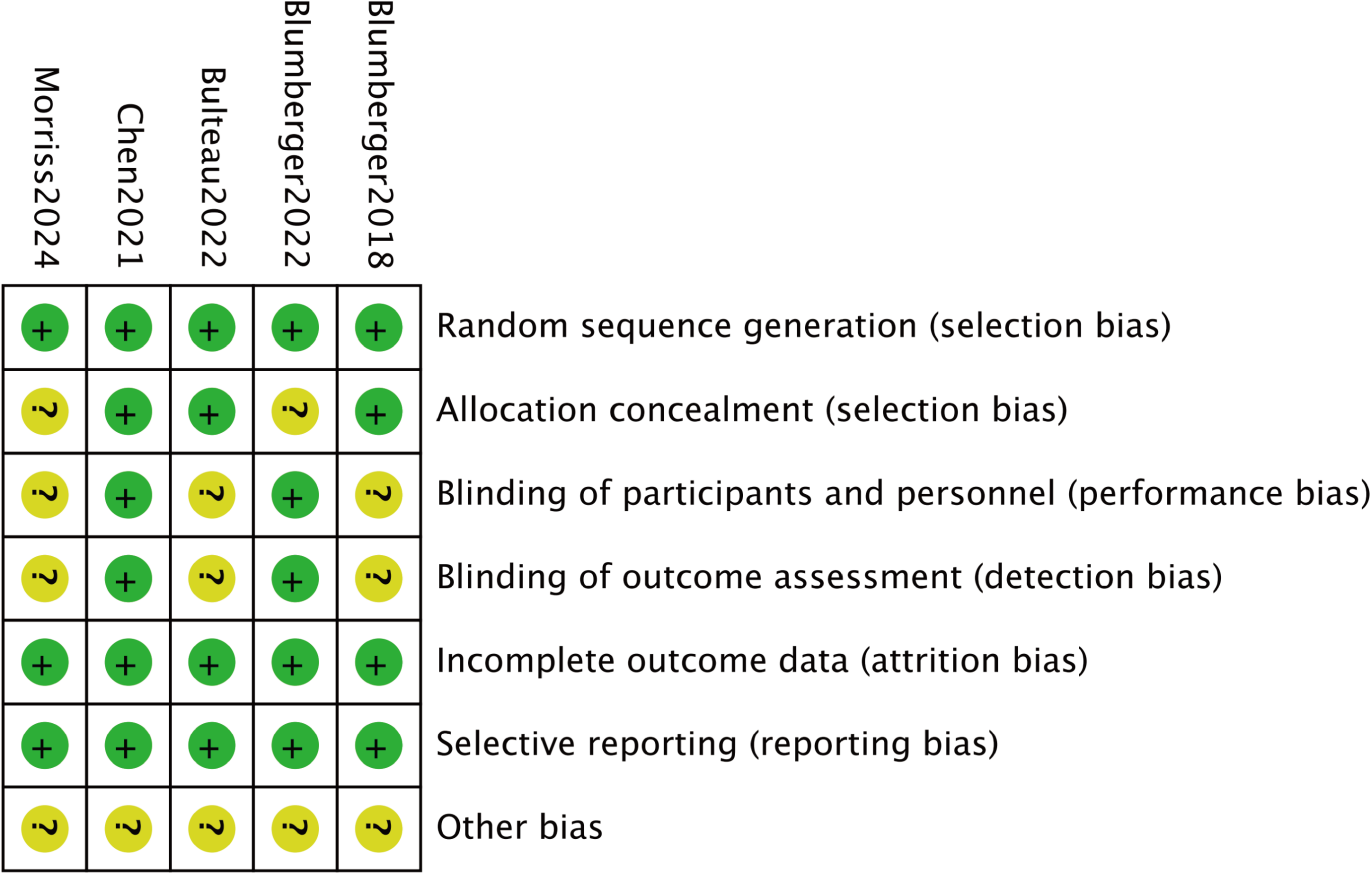
Bias risk summary.

**Figure 3:**
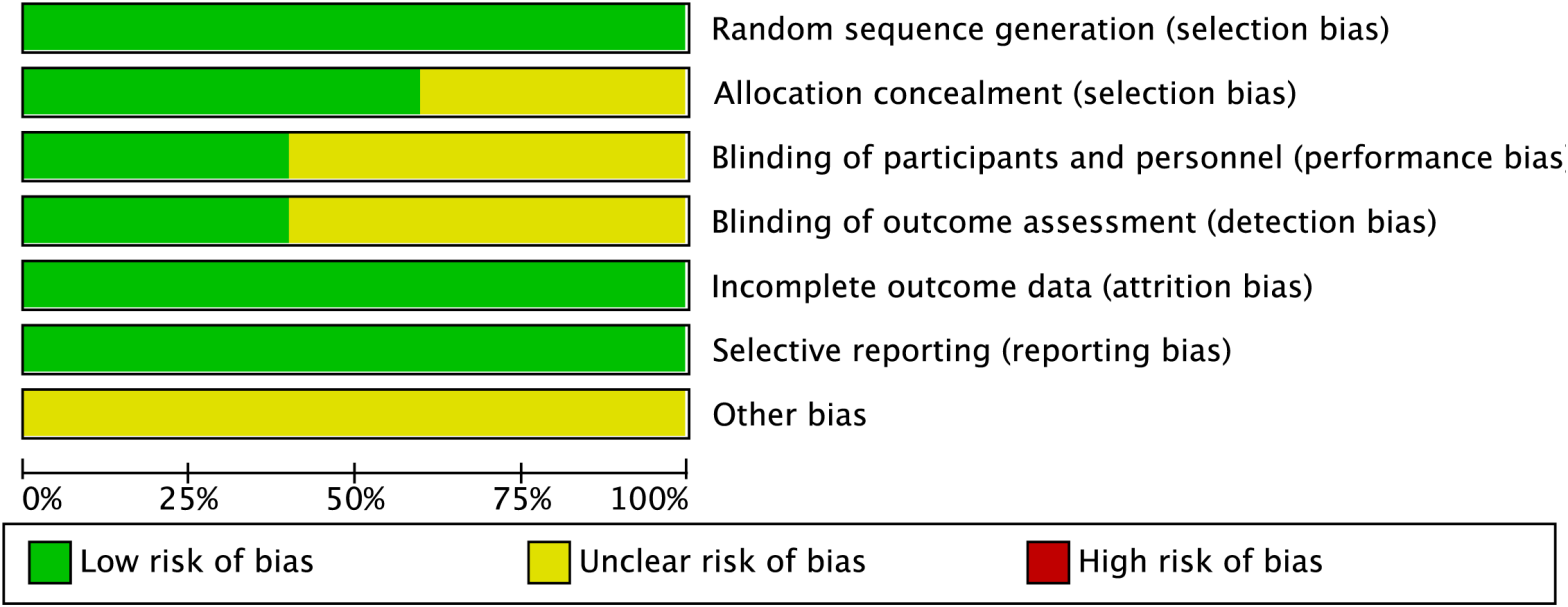
Bias risk graph.

A total of 1,196 participants, aged 41.6 to 61.7 years, were involved in the five investigations. The TBS group included 663 participants, while the rTMS group had 553. TBS was administered at a frequency of 50 Hz, and rTMS at 10 Hz. **Table 1** provides information on the baseline characteristics of the included studies. All studies described the randomization procedures used, although some did not fully detail the blinding strategies. **Figures 2** and **3** present the risk of bias for each study.

**Table 1:** Baseline Characteristics and Methodological Quality of Included Studies.

Study	Year	Country	Sample size		Gender(M/F)	Mean age		Intervention		Outcome
			rTMS	TBS		rTMS	TBS	rTMS	TBS	
Blumberger	2022	Canada	87	85	80/92	67.1	66.3	10HZ	50HZ	F1; F2; F3
Blumberger	2018	Canada	205	209	168/246	43.2	41.6	10HZ	50HZ	F1; F2; F3
Bulteau	2022	France	30	30	19/41	48.5	56.1	10HZ	50HZ	F1;
Chen	2021	Australia	84	211	103/192	48.5	48.67	10HZ	50HZ	F1; F3
Morriss	2024	UK	127	128	123/132	43.8	43.7	10HZ	50HZ	F1; F2; F3

rTMS, repetitive transcranial magnetic stimulation; TBS, Theta burst stimulation; M/F, Male/female; F1, depression; F2, adverse events; F3, anxiety.

### 3.3 Meta-analysis results

#### 3.3.1 Depression scores

**Figure 4:**
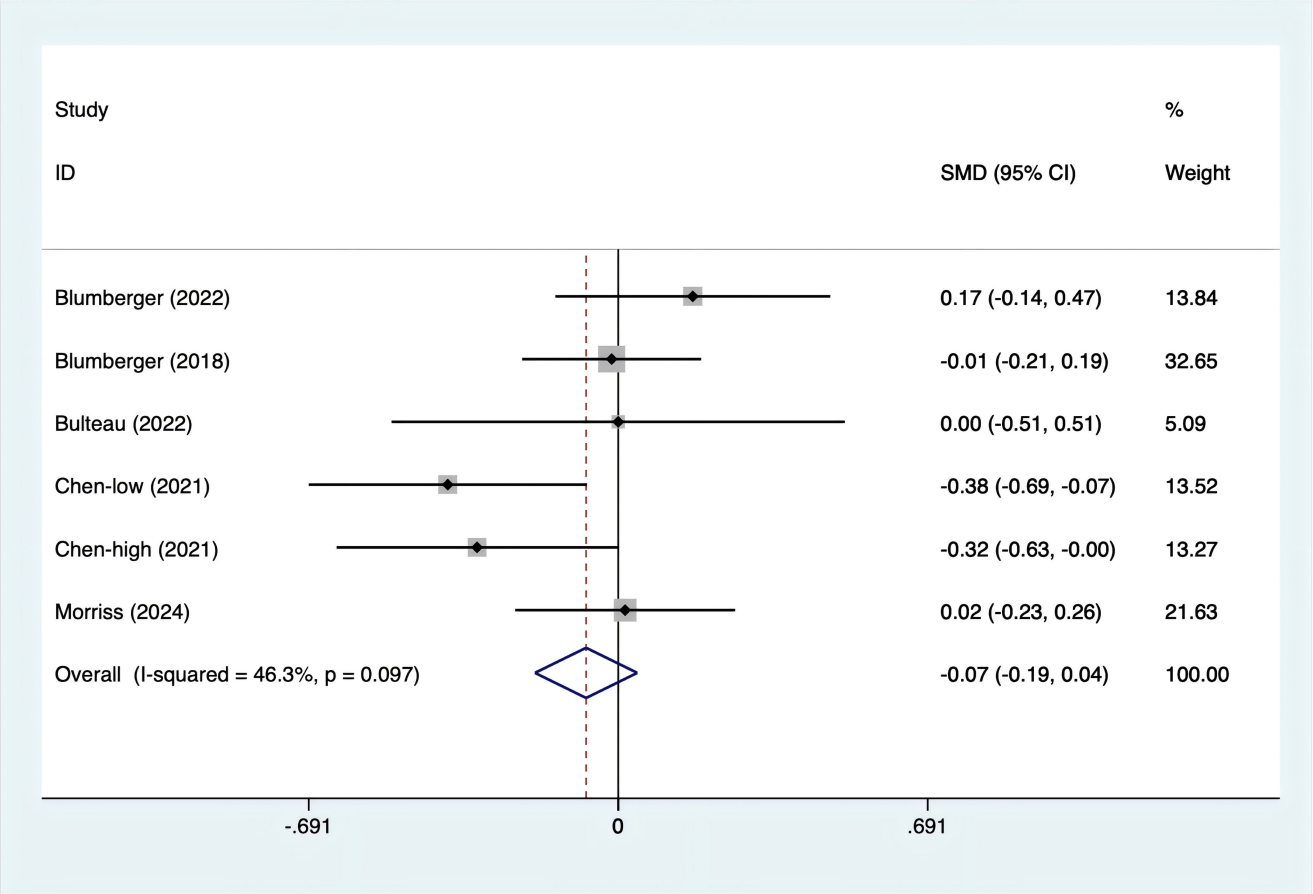
Forest plot of rTMS and TBS in depression scores.

All five studies reported depression scores. Since the test for heterogeneity (I^2^ = 46.3%, p = 0.097) indicated moderate heterogeneity, a fixed-effects model was utilized. The analysis (**Figure 4**) showed no significant difference between rTMS and TBS in terms of depression scores [SMD = -0.07, 95% CI (-0.19, 0.04)].

#### 3.3.2 Anxiety score

**Figure 5:**
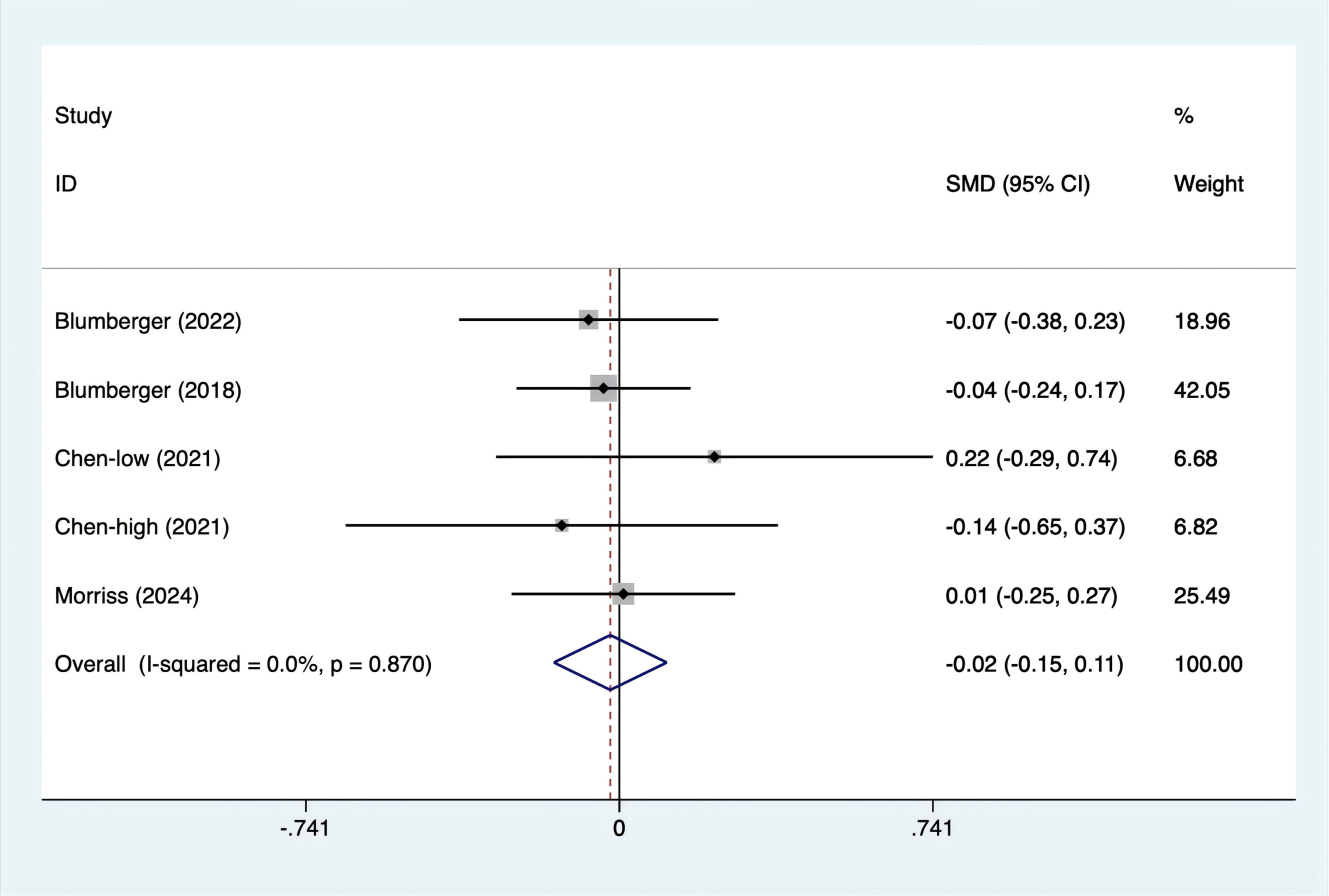
Forest plot of rTMS and TBS in depression scores.

Anxiety scores were reported in four studies. With no heterogeneity detected (I^2^ = 0%, p = 0.870), a fixed-effects model was used. The data (**Figure 5**) revealed no statistically significant difference in anxiety levels between rTMS and TBS [SMD = -0.02, 95% CI (-0.15, 0.11)].

#### 3.3.3 Headache

**Figure 6:**
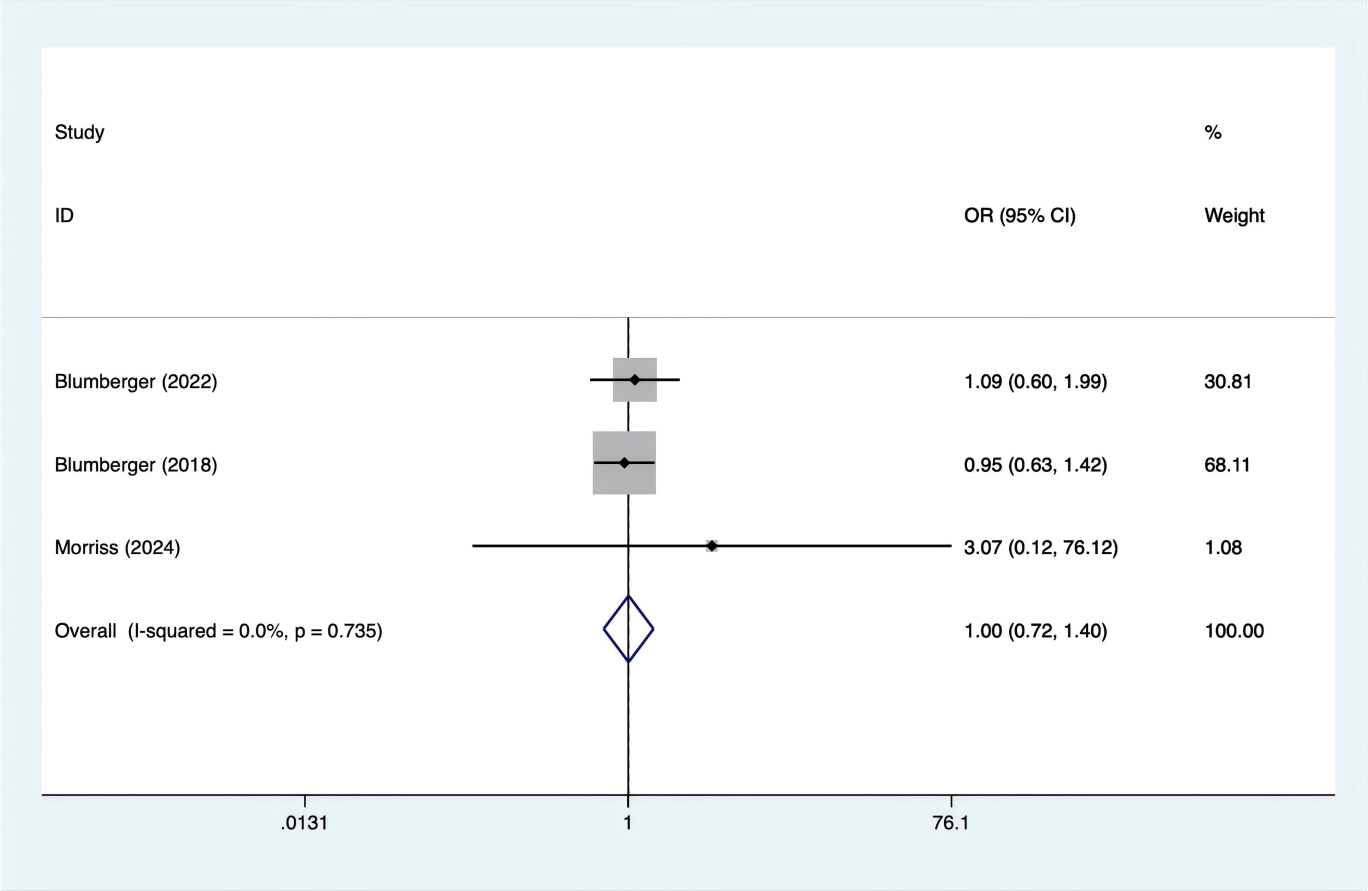
Forest plot of rTMS and TBS in anxiety scores.

Headache incidence was reported in three trials. With no evidence of heterogeneity (I^2^ = 0%, p = 0.735), a fixed-effects model was applied. According to **Figure 6**, there was no significant difference in the occurrence of headaches between rTMS and TBS [OR = 1.00, 95% CI (0.72, 1.40)].

#### 3.3.4 Nausea

**Figure 7:**
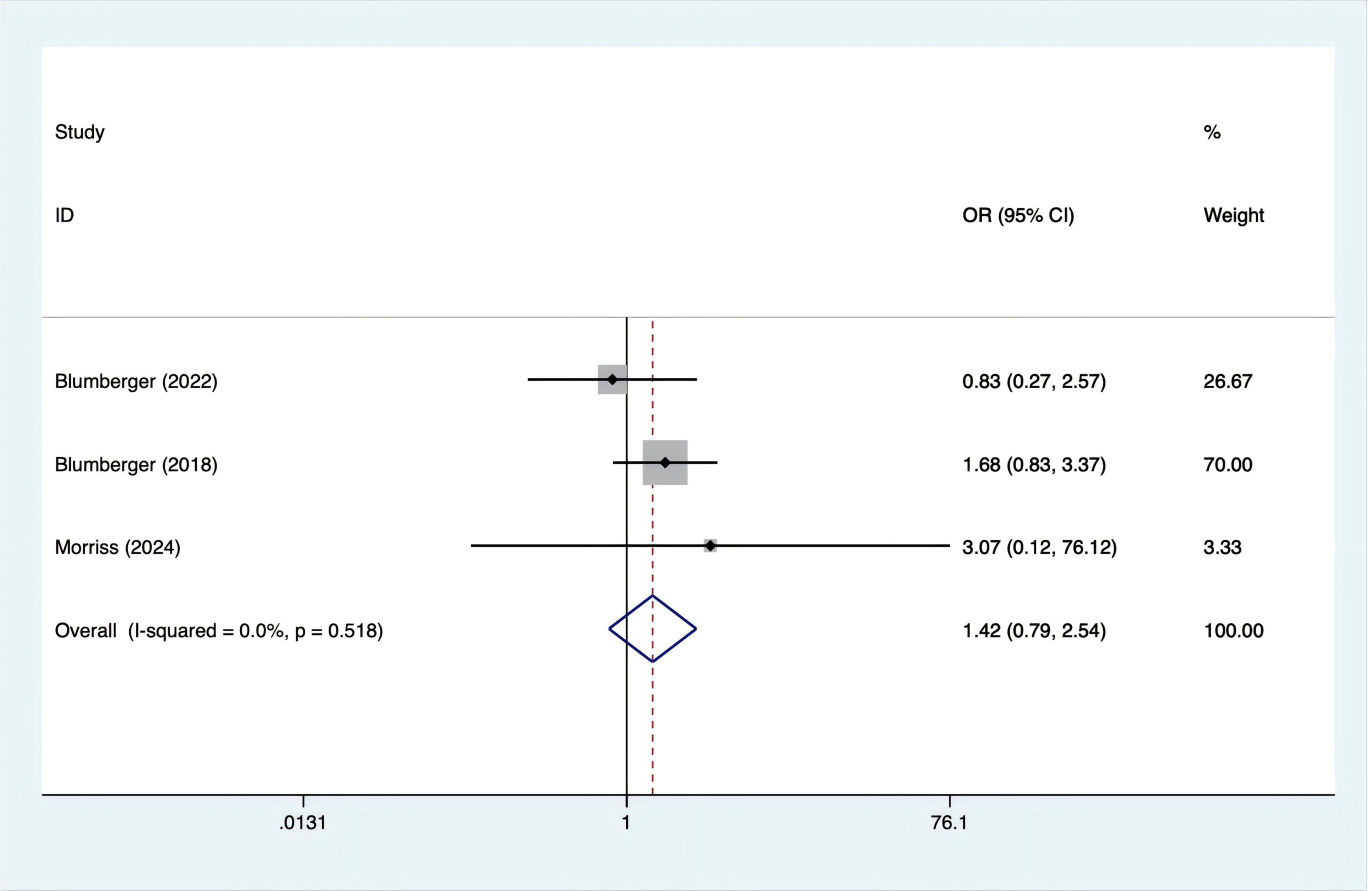
Forest plot of rTMS and TBS in the incidence of nausea.

Three studies reported nausea incidence. Since there was no heterogeneity (I^2^ = 0%, p = 0.518), a fixed-effects model was used. The analysis (**Figure 7**) showed no significant difference in the occurrence of nausea between rTMS and TBS [OR = 1.42, 95% CI (0.79, 2.54)].

#### 3.3.5 Fatigue

**Figure 8:**
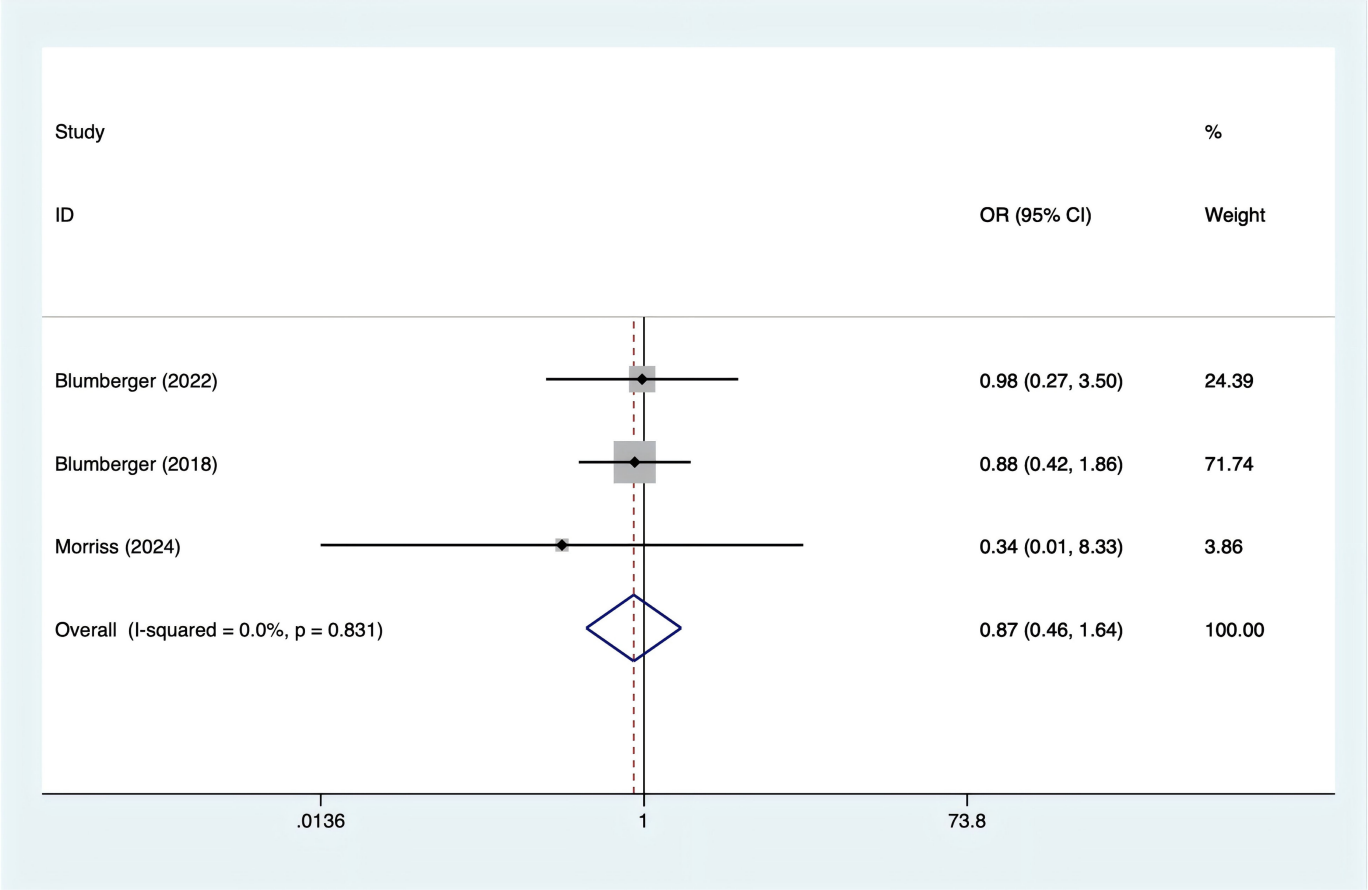
Forest plot of rTMS and TBS in the occurrence of fatigue.

Three studies reported on fatigue. Based on the heterogeneity test results (I² = 0%, p = 0.831), a fixed-effects model was applied. The analysis found no significant difference in fatigue between rTMS and TBS (**Figure 8**; OR = 0.87, 95% CI (0.46, 1.64)).

### 3.4 Publication bias

Egger’s test was used to assess publication bias. The results indicated no significant publication bias across the following categories: depression (p = 0.680), anxiety (p = 0.635), headache (p = 0.125), nausea (p = 0.991), and fatigue (p = 0.436).

## 4 Discussions

This meta-analysis is the first to evaluate both the safety and efficacy of rTMS compared to TBS in the treatment of TRD. Our results revealed no significant differences in the incidence of headaches, nausea, and fatigue, nor in the depression and anxiety scores between rTMS and TBS. These findings suggest that the 37.5-minute, 10 Hz rTMS protocol may not be as effective as the 3-minute intermittent TBS (iTBS) strategy for treating TRD.

rTMS is a treatment method that uses focused magnetic field pulses to directly stimulate the left dorsolateral prefrontal cortex (DLPFC) with a 10 Hz frequency. It has been shown to be a well-tolerated, evidence-based treatment widely used for TRD (26). Theta burst stimulation (TBS) is a non-invasive brain stimulation technique aimed at modulating the underlying neural networks in psychiatric and neurological disorders. TBS can be applied in either intermittent or continuous forms (27). TBS utilizes patterned burst stimulation, requiring only a fraction of the time compared to traditional protocols (28). Compared to standard transcranial magnetic stimulation, TBS may offer a more effective form of physiological stimulation, as it is based on the coupling of brain γ and θ frequency rhythms (20).Additionally, patient-specific factors, such as affective temperament traits, have been shown to influence treatment outcomes in psychiatric disorders, including TRD. Recent studies highlight the role of temperaments as stable, genetically determined predispositions that can modulate clinical dimensions such as disease course, treatment adherence, and therapeutic response (29). For instance, cyclothymic and depressive temperaments are associated with poorer adherence and less favorable outcomes in mood disorders, whereas hyperthymic temperament may confer resilience and predict better responses to certain interventions. Understanding the temperamental profiles of TRD patients could help refine treatment strategies and improve personalized care approaches.The conventional 10 Hz rTMS protocol requires longer sessions and typically takes 4–6 weeks to produce significant antidepressant effects. In contrast, TBS is a more time-efficient form of rTMS, offering comparable antidepressant efficacy in a shorter treatment duration (30). Studies have demonstrated that multiple daily sessions of TBS, either accelerated or intensified, can result in clinically meaningful antidepressant effects in fewer treatment days (31). While many studies on accelerated or intensified TBS have focused on patients with TRD, the subjects in this trial were experiencing their first episode of depression. Early and rapid improvement of clinical symptoms in these patients may improve treatment adherence, reduce suicide risk, lower relapse rates, and aid in the recovery of social functioning (32). In this study, different TMS modalities, combined with sertraline, were used to treat first-episode depression. The results demonstrated that both intensive TBS and 10 Hz rTMS provided similar clinical efficacy, improving depressive and anxiety symptoms, sleep quality, and cognitive function. Notably, intensive TBS showed greater improvement in executive function. Additionally, both treatments were found to be safe and well-tolerated.

Several studies (33, 34) have reported that iTBS offers similar antidepressant efficacy to 10 Hz rTMS, and our findings are consistent with these results. Additionally, two RCTs that tailored and expedited either rTMS or iTBS based on the functional connectivity between the subgenual anterior cingulate cortex (ACC) and the left dorsolateral prefrontal cortex (lDLPFC) demonstrated more substantial reductions in depressive symptoms over a 3–4 week period compared to conventional or sham TBS (35, 36). This suggests that targeting the lDLPFC may be critical for the effectiveness of TMS in treating depression (37). Another study (38) comparing twice-daily TBS with once-daily TBS found no significant difference in antidepressant efficacy after one week of treatment. However, by the end of the 12-week observation period, twice-daily TBS showed superior antidepressant effects, indicating that increasing the frequency of treatments may not result in immediate improvement, but the benefits of intensive TBS may emerge over time.

Previous research has shown that high-frequency rTMS has anxiolytic effects in patients with depression and co-occurring anxiety symptoms (39). We also observed that intensive TBS can alleviate anxiety symptoms. Some studies suggest that rTMS targeting the medial prefrontal cortex and dorsal ACC may help manage anxiety (40). Further research using neuroimaging and electrophysiological techniques is needed to clarify the precise mechanisms by which rTMS targeting the dorsolateral prefrontal cortex improves both anxiety and depression in individuals with concurrent anxiety symptoms.

This study has several limitations. First, the inclusion of only five RCTs limits the generalizability of the findings, reducing statistical power and increasing the risk of errors. Future research should aim to include more studies with larger sample sizes to strengthen the evidence. Second, heterogeneity may have arisen from differences in intervention sites, timings, protocols (e.g., dosages, frequencies), and patient populations. The variability in intervention protocols, such as differences in stimulation frequency and treatment duration, is a significant limitation that warrants more detailed discussion in future studies. Such heterogeneity could impact the interpretation of the results, as different treatment parameters may lead to varying outcomes. To address this, future studies should standardize these factors and conduct sensitivity analyses to assess their impact. Third, subgroup analyses were not feasible due to the limited number of studies. Larger, multicenter RCTs with adequate power are needed to enable meaningful subgroup analyses and gain a deeper understanding of treatment effects in specific patient groups. Additionally, the study did not evaluate the potential protective role of routine psychotherapy and counseling interventions, which are commonly used by patients with depression to prevent or alleviate symptoms. In conclusion, while this study provides valuable insights, addressing the limitations of small sample size, intervention protocol heterogeneity, and the lack of assessment of protective factors in future high-quality, multicenter RCTs will be crucial to confirm these findings and provide stronger clinical evidence.

## 5 Conclusions

While our study did not identify significant differences between rTMS and TBS in terms of depression, anxiety levels, or side effects, TBS offers advantages in terms of shorter session duration and efficiency. With each TBS treatment lasting only 192 seconds, it may be a more affordable option for patients. Therefore, we recommend TBS as a potential therapeutic approach for depression that does not respond to conventional treatments. However, due to the limitations of our research, further high-quality, multicenter randomized controlled trials are necessary to strengthen the evidence supporting this recommendation.

## Data Availability

The original contributions presented in the study are included in the article/**Supplementary Material**. Further inquiries can be directed to the corresponding author.
