# Supplementary material for: A meta-analysis comparing the effectiveness and safety of repetitive transcranial magnetic stimulation versus theta burst stimulation for treatment-resistant depression
Source: Front Psychiatry. 2025 Feb 3;15:1504727. doi: 10.3389/fpsyt.2024.1504727 (PMC11841443; doi:10.3389/fpsyt.2024.1504727)
Supplement: Supplementary file 1 [file Table1.docx]

Table S1 Search strategy

(((((theta burst stimulation[Title/Abstract]) OR (TBS[Title/Abstract])) OR (theta burst transcranial magnetic stimulation[Title/Abstract])) OR (transcranial theta burst stimulation[Title/Abstract])) AND (("Transcranial Magnetic Stimulation"[Mesh]) OR (((((((((Transcranial Magnetic Stimulation[Title/Abstract]) OR (Magnetic Stimulations, Transcranial[Title/Abstract])) OR (Magnetic Stimulation, Transcranial[Title/Abstract])) OR (Stimulations, Transcranial Magnetic[Title/Abstract])) OR (Stimulation, Transcranial Magnetic[Title/Abstract])) OR (Transcranial Magnetic Stimulations[Title/Abstract])) OR (Transcranial Magnetic Stimulation, Paired Pulse[Title/Abstract])) OR (Transcranial Magnetic Stimulation, Repetitive[Title/Abstract])) OR (Transcranial Magnetic Stimulation, Single Pulse[Title/Abstract])))) AND (("Depressive Disorder, Treatment-Resistant"[Mesh]) OR ((((((((((((((((((((((Depressive Disorder, Treatment-Resistant[Title/Abstract]) OR (Depressive Disorders, Treatment-Resistant[Title/Abstract])) OR (Depressive Disorder, Treatment Resistant[Title/Abstract])) OR (Disorders, Treatment-Resistant Depressive[Title/Abstract])) OR (Disorder, Treatment-Resistant Depressive[Title/Abstract])) OR (Treatment-Resistant Depressive Disorder[Title/Abstract])) OR (Treatment-Resistant Depressive Disorders[Title/Abstract])) OR (Treatment Resistant Depression[Title/Abstract])) OR (Depressions, Treatment Resistant[Title/Abstract])) OR (Depression, Treatment Resistant[Title/Abstract])) OR (Resistant Depressions, Treatment[Title/Abstract])) OR (Resistant Depression, Treatment[Title/Abstract])) OR (Treatment Resistant Depressions[Title/Abstract])) OR (Refractory Depression[Title/Abstract])) OR (Depression, Refractory[Title/Abstract])) OR (Depressions, Refractory[Title/Abstract])) OR (Refractory Depressions[Title/Abstract])) OR (Therapy-Resistant Depression[Title/Abstract])) OR (Depressions, Therapy-Resistant[Title/Abstract])) OR (Depression, Therapy-Resistant[Title/Abstract])) OR (Therapy Resistant Depression[Title/Abstract])) OR (Therapy-Resistant Depressions[Title/Abstract])))
